# Class III Malocclusion in Growing Patients: Facemask vs. Functional Appliance: Experimental Study

**DOI:** 10.3390/bioengineering12101027

**Published:** 2025-09-26

**Authors:** Lucia Giannini, Guido Galbiati, Cinzia Maspero, Gianna Dipalma, Roberto Biagi

**Affiliations:** 1 Dipartimento di Scienze Biomediche, Chirurgiche e Odontoiatriche, Università degli Studi di Milano, 20122 Milan, Italy; lucia.giannini@unimi.it (L.G.); roberto.biagi@unimi.it (R.B.); 2 Fondazione IRCCS Cà Granda Ospedale Maggiore Policlinico, 20122 Milan, Italy; 3 Department of Interdisciplinary Medicine, University of Bari “Aldo Moro”, 70121 Bari, Italy; gianna.dipalma@uniba.it

**Keywords:** orthodontics appliances, postero-anterior facemask, functional appliance

## Abstract

**Objective:** We compared the skeletal effects of postero-anterior facemask (PAF) and functional appliance (FA) therapy in growing patients with Class III malocclusion. **Materials and Methods:** A total of 85 patients (mean age 9 ± 0.2 years) were treated with either a PAF (n = 50) or a FA (n = 35). Pre- and post-treatment cephalometric records were analyzed to assess sagittal (SNA, SNB, ANB, Wits appraisal) and vertical changes. Treatment outcomes were compared using Student’s *t* test for paired samples. **Results:** PAF therapy produced significantly greater improvements in the ANB angle (mean increase 4.1° vs. 1.7°) and Wits appraisal (2.4 mm vs. 0.9 mm) compared to FAs. Vertical control was superior in the PAF group, which showed a reduction in lower facial height, whereas FA patients exhibited a slight increase. **Conclusions:** PAF therapy was more effective than FAs in improving both sagittal and vertical skeletal relationships in growing Class III patients. Functional appliances provided only modest skeletal effects, mainly influencing mandibular position. Early intervention with a PAF should be considered the treatment of choice when maxillary protraction and vertical control are required.

## 1. Introduction

Class III malocclusion is characterized by a skeletal and/or dental sagittal discrepancy in the relationship between the maxilla and mandible. It often leads to functional disturbances and compromised aesthetics [1].

This malocclusion is usually associated with mandibular prognathism or maxillary hypoplasia or both and can lead to occlusal problems, speech difficulties, and an altered facial appearance, all of which can have significant psychological and social impacts, including in growing children [2,3,4,5].

In growing patients, an early intervention can yield more favorable outcomes, both in females and in males: in fact, during growth, the maxilla and mandible are responsive to orthopedic forces and a correct treatment can lead to a functional and esthetic success [6,7,8,9].

There are two main therapeutic approaches that are used to treat Class III malocclusions in growing patients: postero-anterior facemask (PAF) therapy and functional appliances (FAs) [10,11].

The postero-anterior facemask promotes repositioning of the maxilla in a forward direction, while simultaneously controlling mandibular growth. It is effective in managing Class III malocclusions in growing patients, particularly in those with maxillary deficiency. This is a skeletal modification of the anterior growth of the maxilla with the use of reverse traction and a face mask. Mandibular excess has been widely discussed in the literature and several authors have described the advantages and limits of this skeletal treatment [9,10,11].

In contrast, Class III functional appliances are designed to alter the position of the mandible. These appliances work by encouraging mandibular repositioning in response to the backward functional orthopedic forces applied during therapy. While functional appliances can produce favorable changes in occlusion and mandibular posture, they tend to have a more limited effect on the forward positioning of the maxilla: the main action of these appliances consists of modifying the functional position of the mandible rather than changing the maxilla [12].

The effectiveness of these two treatment approaches has been widely discussed in the literature: previous studies suggest that PAF therapy tends to induce more significant skeletal changes in the maxilla, leading to greater improvements in the sagittal skeletal relationship. In contrast, functional appliances are often seen as more effective in modifying the dental and mandibular components, but they may be less effective in skeletal corrections [13,14].

Although both treatments have shown success in treating Class III malocclusion in growing patients, the comparative effectiveness, particularly in terms of their effects on the sagittal and vertical dimensions, has not been deeply examined yet [13].

This study aimed to compare the outcomes of two alternative treatment modalities for Class III malocclusion: functional appliances and facemask therapy. The sagittal and vertical skeletal changes induced by each treatment modality and the examination of the role of patient compliance, treatment duration, and post-treatment stability were evaluated.

## 2. Materials and Methods

This study was conducted on a sample of 85 patients with a mean age of 9 ± 0.2 years old, presenting a Class III dento-skeletal malocclusion treated at Fondazione IRCCS Ca’ Granda, Ospedale Maggiore Policlinico Milan between January 2023 and August 2024.

In this study, all patients diagnosed with Class III malocclusion were offered therapy with a postero-anterior facemask.

However, some parents of young patients declined this treatment due to the perceived invasiveness of the device.

For these patients, an alternative suggestion was made to undergo therapy with a functional appliance rather than simply waiting for the end of skeletal growth. The FA used was a customized Class III acrylic (orthodontic laboratory of the Milan Polyclinic, University of Milan, Milan, Italy) appliance with anterior bite blocks and posterior cupping, designed to promote mandibular repositioning and discourage anterior functional shifts. The appliances were constructed according to the design principles of the Frankel III, with a simplified structure aimed at enhancing comfort and compliance.

Wear instructions included full-time usage (at least 16 h/day), including during sleep, with removal only during meals and oral hygiene.

This choice formed the basis of the present study. We know that the sample is unbalanced, as, under similar conditions, the majority of patients underwent therapy with the postero-anterior facemask. Nonetheless, this study provides an objective comparison, although with a smaller sample, of the differential effects of the two treatment approaches.

The patients were divided into two treatment groups: 50 patients underwent therapy with a PAF, while 35 patients opted for functional therapy with a Class III FA.

The PAF therapy aimed to stimulate corrective maxillary growth and improve the sagittal relationship between the maxilla and mandible. In contrast, the functional appliance therapy aimed to modulate the mandibular position without direct traction.

This study was approved by the Research Protocol of the Fondazione IRCCS Ca’Granda, Ospedale Maggiore Policlinico (Ethical Committee: 7597-Prot. 0015641—16/02/23).

The study was conducted in accordance with the principles of good clinical practice (ICH/ISO 14155) and the Helsinki Declaration (2008) [7]. Patients’ parents or their guardians signed an informed consent form, allowing us to use diagnostic records for research motivations.

Records consisted of lateral cephalograms obtained before and after therapy of patients that underwent orthodontic treatment at the Department of Biomedical Surgical and Dental Sciences.

The inclusion criteria for the study were:

- Diagnosis of Class III dento-skeletal malocclusion.
- Pre- and post-treatment radiographs X rays.
- Age between 8 and 10 years at the start of treatment.
- Adequate cooperation during the treatment.

Exclusion criteria were:

- Lack of complete and correct radiographic documentation.
- Insufficient compliance with treatment protocols.
- Systemic or growth diseases that could influence the results.
- Previous orthodontic treatment.

Treatment efficacy was evaluated using pre- and post-treatment cephalometric lateral analysis following School of Milan, measuring the following parameters: SNA (angle between sella-nasion line and nasion-A line), SNB (angle between sella-nasion line and nasion-B line), ANB (difference between SNA and SNB angles), the distance between point A and point B on the occlusal plane (Wits appraisal) and the distance between point SNA and ME [15]. The software used was Ceph Pratic. The lateral projection teleradiographs of the skull were performed at the radiology department of the Fondazione IRCCS Ca’Granda, Ospedale Maggiore Policlinico by the same operator.

Measurements were performed using cephalometric software to ensure accuracy and repeatability and were repeated by two different operators and the average was considered to minimize individual variability.

Method error was calculated using Dahlberg’s formula to assess measurement reliability. Treatment stability was evaluated 12 months after completion.

Significant differences between the groups were analyzed using Student’s *t* test for paired samples to compare improvements in cephalometric parameters between patients treated with postero-anterior facemask and those treated with functional appliances.

The sample size was calculated based on an alpha significance level of 5% and beta of 20% to achieve 80% of power to detect a mean difference of 2.5 mm with a standard deviation of 2 mm in Wits appraisal change between pre and posttreatment. The sample size calculation showed that a sample size of 11 patients was needed.

## 3. Results

The results of the study demonstrated a statistically significant difference in the treatment results between the two groups: those treated with the postero-anterior facemask (PAF) and those treated with the functional appliance (FA).

**Figure 1:**
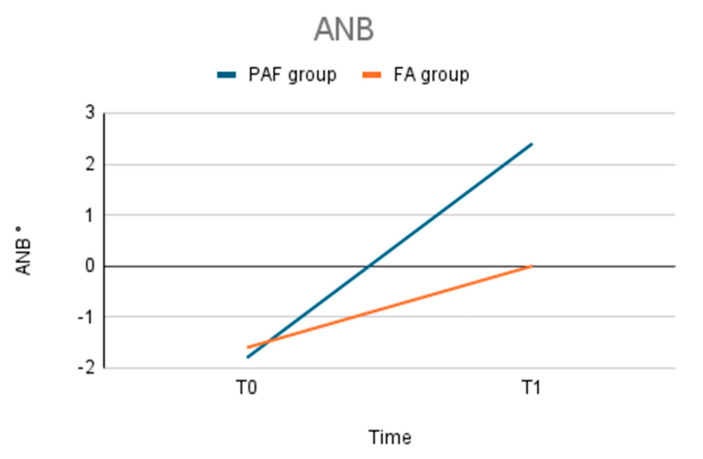
ANB angle: changes during the treatment. Comparison between the two groups.

The PAF group showed a greater improvement in cephalometric parameters associated with sagittal correction, especially in the ANB angle and the distance between points A and B (Figure 1, Table 1). The SNB angle varies more significantly in the FA group.

In fact, the functional appliance therapy aimed to modulate the mandibular position without direct traction.

**Figure 2:**
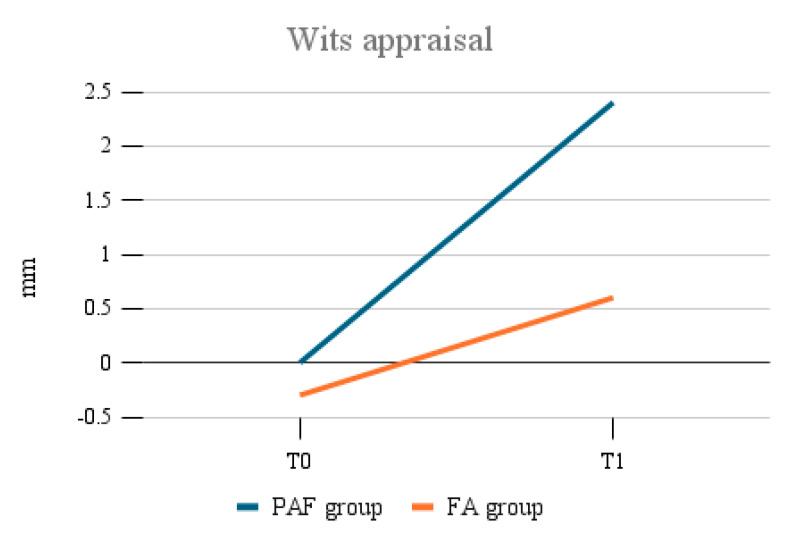
Wits appraisal index: changes during the treatment. Comparison between the two groups.

The average increase in the ANB angle was 4.1° (±1.5), compared to 1.7° (±0.8) in the FA group. Similarly, the Wits appraisal index increased by an average of 2.4 mm (±1.2) in the PAF group, whereas the FA group showed a lower increase of 0.9 mm (±0.5) (Figure 2).

The statistical analysis confirmed these differences were significant (*p* < 0.05): the postero-anterior facemask treatment resulted in a more substantial correction of sagittal discrepancies.

**Figure 3:**
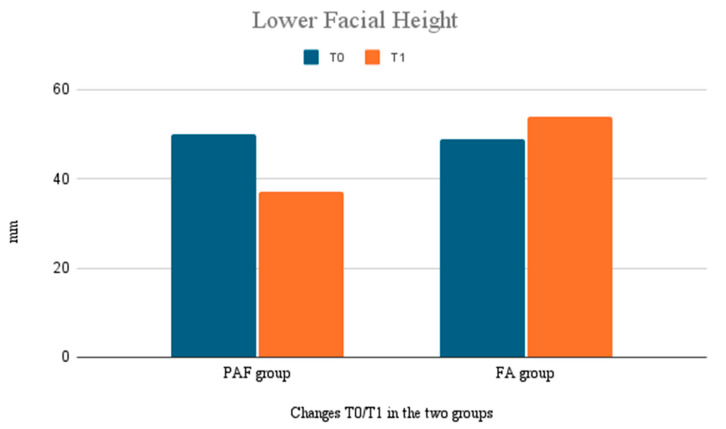
Lower Facial Height: changes during the treatment. Comparison between the two groups.

The changes in the vertical dimension were also evaluated (Figure 3).

The PAF group showed a significant reduction in vertical growth, with a decrease in lower facial height of 1.3 mm (±0.9), which has the advantage of preventing excessive vertical growth often seen in Class III cases. The FA group showed a slight increase in the vertical dimension, with an average increase of 0.5 mm (±0.4).

This difference was statistically significant (*p* < 0.05), highlighting that the PAF was more effective at controlling vertical growth, while the FA has a more limited effect on the posterior maxillary region.

The FA group demonstrated greater intragroup variability in results, especially in terms of sagittal correction, which may reflect lower patient compliance with appliance use.

## 4. Discussion

This study aimed to evaluate the effects of PAF and functional therapy with a Class III functional appliance to treat Class III dento-skeletal malocclusion in growing patients. (FA) [16,17]. The results indicate that PAF therapy led to significantly greater improvements in both the sagittal and vertical dimensions compared to the FA group.

The results obtained underscore the importance of choosing the appropriate treatment modality based on the severity of the malocclusion, the growth pattern of the patient, and their ability to comply with the recommended appliance wear. PAF treatment proved more effective than FA treatment in both sagittal and vertical planes, and also offers greater long-term stability, making it a preferred option for achieving stronger corrections [16,17].

On the other hand, the FA remains a valuable choice, in particular for patients with less important discrepancies or those who may not be suitable candidates for the more intensive PAF therapy. Additionally, the ease of use and comfort of the FA may still appeal to certain patients.

The increased variability within the FA group, which may be attributable to lower compliance with appliance use, represents a critical factor in treatment planning, since reduced adherence could limit the ability of functional appliances to deliver consistent and durable results.

The FA group showed a higher degree of variability in the clinical outcomes, which may be linked to inconsistent usage patterns so the facemask, although uncomfortable, proved to be a more effective option for correcting skeletal class III malocclusions.

Our findings are similar to previous studies that have demonstrated the effectiveness of PAFs in treating Class III malocclusions, offering significant advancements over functional appliance therapy [18,19,20].

The greater correction in the PAF group could be attributed to the more direct mechanical forces applied by the facemask, which likely exerted a stronger influence on the maxilla, promoting forward movement in the sagittal plane.

The results obtained in the vertical dimension suggest that while the FA group shows some improvements, it may not be as effective in controlling vertical-dimension changes, which is critical for achieving optimal facial aesthetics in growing patients. Also, these results are consistent with prior studies that emphasize the ability of the PAF to manage vertical growth patterns in Class III malocclusion [21].

The importance of patient cooperation is underlined in several studies in the literature, which may explain why the FA group, despite showing some improvements, experienced less consistent outcomes [22,23,24].

The PAF group exhibited a more substantial improvement in the ANB angle and the sagittal relationship between the maxilla and mandible, which was reflected in the increased distance between points A and B. These results are aligned with previous studies, which found similar improvements in sagittal relationships with PAF therapy, confirming that this method effectively induces maxillary protraction and corrects Class III malocclusions in growing patients [25].

Consistent with our results, Ngan (1998) demonstrated that postero-anterior facemask therapy represents one of the most effective modalities for enhancing the sagittal skeletal relationship, especially when applied at an early stage of growth [26].

According to Baccetti (1998), the combination of a bonded maxillary expander and facemask therapy yields greater effectiveness when applied during early mixed dentition than in the late phase, especially with respect to maxillary protraction [27].

In contrast, the FA group showed more modest improvements in sagittal correction probably because functional appliance primarily works by modifying the position of the mandible, rather than inducing skeletal changes in the maxilla. The lower correction of the ANB angle and Wits appraisal in FA group is similar with the result obtained by some authors, who noted that functional appliances are more effective in modifying the mandibular position rather than significantly altering maxillary development, thus resulting in more modest sagittal changes compared to other modalities like the postero-anterior facemask. The greater variability in outcomes among the FA group, probably due to patient compliance, was observed also by previous studies, which reported that the success of functional appliance therapy is often compromised by inconsistent appliance wear, leading to less predictable results [28].

As for the vertical dimension, our study indicates that PAF therapy led to a slight decrease in the vertical growth of the lower facial height, which is often beneficial in cases of excessive mandibular growth. This reduction in the vertical dimension may stem from the combined effects of maxillary protraction and mandibular control, both typical of postero-anterior facemask therapy. Our results are in agreement with previous study, which discovered that postero-anterior facemask therapy can reduce the vertical dimension, aiding in the management of excessive vertical growth in Class III patients. In contrast, the FA group experienced a slight increase in the vertical dimension, with an average rise in lower facial height [29]. This finding corresponds with previous studies, which noted that functional appliances often encourage a slight increase in vertical growth due to their action on the mandible, which may influence the vertical dimension [30].

Both treatments showed positive effects; however, the PAF group exhibited greater stability over time, experiencing fewer losses in the corrections achieved after treatment. This increased stability is likely attributed to the more significant skeletal changes brought about by the facemask therapy, which are less likely to relapse compared to the dental and soft tissue changes associated with the functional appliance. This finding aligns with Ngan & Hägg (1998), who observed that the effects of skeletal treatments like PAFs are generally more stable than those of purely functional therapies, which tend to be more vulnerable to relapse once treatment ends [26]. Additionally, the enhanced stability of the PAF group is in line with previous studies, which pointed out that the long-term effects of postero-anterior facemask therapy are more enduring compared to other non-skeletal treatments [26,30,31].

While no significant complications were noted in either group, the FA group showed more variability in results, probably because of differences in patient compliance. This highlights how crucial patient cooperation is for the success of functional therapy [32,33,34,35,36]. The method error analysis validated the reliability of our cephalometric measurements, and we reduced inter-operator variability by averaging all measurements.

Our study has several strengths. Firstly, it compares two commonly used treatment methods for Class III malocclusion, providing important insights into their effectiveness for patients who are still growing [37,38,39,40]. The use of pre- and post-treatment cephalometric analysis guarantees that the results are evaluated in an objective and quantitative manner. Furthermore, examining changes in the vertical dimension is a significant addition, as this factor is frequently neglected in other research that primarily concentrates on sagittal correction.

This study also has some limitations. A key limitation is the small number of patients in the FA group (n = 35), which may restrict the applicability of the findings to a wider population. Additionally, the patients who opted for functional appliance therapy may represent a subgroup with distinct characteristics, such as lower compliance or milder malocclusions, potentially introducing bias into the results. A larger and more balanced sample size would help address this limitation.

One drawback is the retrospective design of the study, which might introduce selection bias. The choice to pursue PAF therapy or FA therapy was made by the patients and their families, potentially leading to confounding factors like personal preferences or the severity of malocclusion. To overcome these biases and achieve a more robust comparison of the two treatment modalities, a randomized controlled trial would be necessary.

The follow-up period for the study was relatively short, lasting only 1 year after treatment. A longer follow-up is crucial to evaluate treatment stability of the treatment results and to identify any long-term differences between the two groups, especially regarding relapse or recurrence of malocclusion.

While our study supports the use of postero-anterior facemask therapy as a more effective and stable treatment option for correcting Class III dento-skeletal malocclusion in growing patients, particularly when sagittal correction and vertical control are essential goals, the limitations of the study suggest that further research with larger sample sizes, longer follow-up periods, and randomized controlled designs is needed to confirm these findings and to evaluate their long-term stability.

## 5. Conclusions

Postero-anterior facemask therapy produced greater improvements in sagittal and vertical skeletal relationships than functional appliance therapy in growing Class III patients. The PAF was more effective in maxillary protraction and vertical control, while FAs mainly influenced the mandibular position with more variable outcomes. Although both approaches can gain positive results, PAFs should be preferred when significant skeletal correction is required. The choice between a PAF and an FA should be individualized according to clinical findings, patient motivation, compliance, and family preferences. Future studies with larger samples and longer follow-up would help to further support these findings.

## Figures and Tables

**Table 1:** ANB, SNA, and SNB Angles: changes during the treatment. Comparison between the two groups.

	PAF	PAF	FA	FA
	T0	T1	T0	T1
**ANB (°)**	−1.8 ± 0.3	2.4 ± 0.4	−1.6 ± 0.4	0 ± 0.3
**SNA (°)**	77.5 ± 0.2	82 ± 0.3	77.7 ± 0.4	81.4 ± 0.3
**SNB (°)**	79.3 ± 0.4	79.6 ± 0.3	79.3 ± 0.1	81.3 ± 0.2
**WITS (mm)**	0 ± 0.4	2.4 ± 0.3	−0.3 ± 0.3	0.6 ± 0.5
**vertical facial height difference (mm)**	−1.3 ± 0.7		+0.5 ± 0.2	

## Data Availability

The data presented in this study are available on reasonable request, after the signature of a formal data sharing agreement in anonymous form, from the corresponding author because they are protected by privacy.

## References

[1] De Ridder L., Aleksieva A., Willems G., Declerck D., Cadenas de Llano-Pérula M. (2022). Prevalence of Orthodontic Malocclusions in Healthy Children and Adolescents: A Systematic Review. Int. J. Environ. Res. Public Health.

[2] Ding H., Wu J., Zhao W., Matinlinna J.P., Burrow M.F., Tsoi J.K.H. (2023). Artificial intelligence in dentistry—A review. Front. Dent. Med..

[3] Hu J., Jiang Y., Wang D., Guo S., Li S., Jiang H., Cheng J. (2021). Comparison of cost-effectiveness and benefits of surgery-first versus orthodontics-first orthognathic correction of skeletal class III malocclusion. Int. J. Oral Maxillofac. Surg..

[4] Mandall N., Aleid W., Cousley R., Curran E., Caldwell S., DiBiase A., Dyer F., Littlewood S., Nute S., Campbell S.J. (2024). The effectiveness of bone anchored maxillary protraction (BAMP) in the management of class III skeletal malocclusion in children aged 11–14 years compared with an untreated control group: A multicentre two-arm parallel randomised controlled trial. J. Orthod..

[5] Tarraf N.E., Dalci O., Dalci K., Altug A.T., Darendeliler M.A. (2023). A retrospective comparison of two protocols for correction of skeletal Class III malocclusion in prepubertal children: Hybrid hyrax expander with mandibular miniplates and rapid maxillary expansion with face mask. Prog. Orthod..

[6] DiBiase A.T., Seehra J., Papageorgiou S.N., Cobourne M.T. (2022). Do we get better outcomes from early treatment of Class III discrepancies?. Br. Dent. J..

[7] Maspero C., Galbiati G., Giannini L., Farronato G. (2015). Sagittal and vertical effects of transverse sagittal maxillary expander (TSME) in three different malocclusion groups. Prog. Orthod..

[8] Tabellion M., Lisson J.A. (2024). Dentofacial and skeletal effects of two orthodontic maxillary protraction protocols: Bone anchors versus facemask. Head. Face Med..

[9] Maspero C., Galbiati G., Giannini L., Guenza G., Esposito L., Farronato G. (2018). Titanium TSME appliance for patients allergic to nickel. Eur. J. Paediatr. Dent..

[10] Lyu L., Lin H., Huang H. (2022). The effect of combined maxillary pad movable appliance and FR-III functional appliance in the treatment of skeletal Class III malocclusion of deciduous teeth. BMC Oral Health.

[11] Marcílio Santos E., Kalil Bussadori S., Ratto Tempestini Horliana A.C., Moraes Moriyama C., Jansiski Motta L., Pecoraro C., Cabrera Martimbianco A.L. (2023). Functional orthopedic treatment for anterior open bite in children. A systematic review of randomized clinical trials. J. Orofac. Orthop..

[12] Owens D., Watkinson S., Harrison J.E., Turner S., Worthington H.V. (2024). Orthodontic treatment for prominent lower front teeth (Class III malocclusion) in children. Cochrane Database Syst. Rev..

[13] Ngan P., Moon W. (2015). Evolution of Class III treatment in orthodontics. Am. J. Orthod. Dentofacial Orthop..

[14] Farronato G., Rosso G., Giannini L., Galbiati G., Maspero C. (2016). Correlation between skeletal Class II and temporomandibular joint disorders: A literature review. Minerva Stomatol..

[15] Maspero C., Galbiati G., Perillo L., Favero L., Giannini L. (2012). Orthopaedic treatment efficiency in skeletal Class III malocclusions in young patients: RME-face mask versus TSME. Eur. J. Paediatr. Dent..

[16] Caruso S., Lisciotto E., Caruso S., Marino A., Fiasca F., Buttarazzi M., Sarzi Amadè D., Evangelisti M., Mattei A., Gatto R. (2023). Effects of Rapid Maxillary Expander and Delaire Mask Treatment on Airway Sagittal Dimensions in Pediatric Patients Affected by Class III Malocclusion and Obstructive Sleep Apnea Syndrome. Life.

[17] Yang X., Li C., Bai D., Su N., Chen T., Xu Y., Han X. (2014). Treatment effectiveness of Frankel function regulator on the Class III malocclusion: A systematic review and meta-analysis. Am. J. Orthod. Dentofac. Orthop..

[18] Lopatienė K., Trumpytė K. (2018). Relationship between unilateral posterior crossbite and mandibular asymmetry during late adolescence. Stomatologija.

[19] Kan H., Sözen T., Öğretmenoğlu O., Ciğer S. (2024). Evaluation of the Effects of Orthopedic Treatment on the Dentofacial Structure and Upper Airway of Subjects with Skeletal Class III Malocclusion. Turk. J. Orthod..

[20] Caroccia F., Juloski J., Juloski J., Marti P., Lampus F., Vichi A., Giuntini V., Rutili V., Nieri M., Goracci C. (2025). 3D printed customized facemask for early treatment of Class III malocclusion: A two-center case series feasibility study. Minerva Dent. Oral Sci..

[21] Muthukumar K., Vijaykumar N.M., Sainath M.C. (2016). Management of skeletal Class III malocclusion with face mask therapy and comprehensive orthodontic treatment. Contemp. Clin. Dent..

[22] Nahajowski M., Lis J., Sarul M. (2022). Orthodontic Compliance Assessment: A Systematic Review. Int. Dent. J..

[23] Wafaie K., Rizk M.Z., Basyouni M.E., Daniel B., Mohammed H. (2023). Tele-orthodontics and sensor-based technologies: A systematic review of interventions that monitor and improve compliance of orthodontic patients. Eur. J. Orthod..

[24] Shah N. (2017). Compliance with removable orthodontic appliances. Evid. Based Dent..

[25] Foersch M., Jacobs C., Wriedt S., Hechtner M., Wehrbein H. (2015). Effectiveness of maxillary protraction using facemask with or without maxillary expansion: A systematic review and meta-analysis. Clin. Oral Investig..

[26] Ngan P., Yiu C., Hu A., Hägg U., Wei S.H., Gunel E. (1998). Cephalometric and occlusal changes following maxillary expansion and protraction. Eur. J. Orthod..

[27] Baccetti T., McGill J.S., Franchi L., McNamara J.J.A., Tollaro I. (1998). Skeletal effects of early treatment of Class III malocclusion with maxillary expansion and face-mask therapy. Am. J. Orthod. Dentofac. Orthop..

[28] Franchi L., Baccetti T., Tollaro I. (1997). Predictive variables for the outcome of early functional treatment of Class III malocclusion. Am. J. Orthod. Dentofac. Orthop..

[29] Snigdha P., Sumita M. (2016). Treatment of Class III with Facemask Therapy. Case Rep. Dent..

[30] Kılıçoğlu H., Öğütlü N.Y., Uludağ C.A. (2017). Evaluation of Skeletal and Dental Effects of Modified Jasper Jumper Appliance and Delaire Face Mask with Pancherz Analysis. Turk. J. Orthod..

[31] Sarangal H., Namdev R., Garg S., Saini N., Singhal P. (2020). Treatment Modalities for Early Management of Class III Skeletal Malocclusion: A Case Series. Contemp. Clin. Dent..

[32] Farronato G., Giannini L., Galbiati G., Stabilini S.A., Maspero C. (2013). Orthodontic-surgical treatment: Neuromuscular evaluation in open and deep skeletal bite patients. Prog. Orthod..

[33] Maspero C., Galbiati G., Giannini L., Guenza G., Farronato M. (2018). Class II division 1 malocclusions: Comparisons between one- and two-step treatment. Eur. J. Paediatr. Dent..

[34] Lee N.K., Kim S.H., Park J.H., Son D.W., Choi T.H. (2022). Comparison of treatment effects between two types of facemasks in early Class III patients. Clin. Exp. Dent. Res..

[35] Montenegro V., Inchingolo A.D., Malcangi G., Limongelli L., Marinelli G., Coloccia G., Laudadio C., Patano A., Inchingolo F., Bordea I.R. (2021). Compliance of children with removable functional appliance with microchip integrated during COVID-19 pandemic: A systematic review. J. Biol. Regul. Homeost. Agents.

[36] Lena Y., Bozkurt A.P., Yetkiner E. (2017). Patients’ and Parents’ Perception of Functional Appliances: A Survey Study. Turk. J. Orthod..

[37] Liao Y.F., Lu T.C., Chang C.S., Chen Y.A., Chen Y.F., Chen Y.R. (2024). Surgical Occlusion Setup and Skeletal Stability of Correcting Cleft-Associated Class III Deformity Using Surgery-First Bimaxillary Surgery. Plast. Reconstr. Surg..

[38] Maspero C., Galbiati G., Del Rosso E., Farronato M., Giannini L. (2019). RME: Effects on the nasal septum. A CBCT evaluation. Eur. J. Paediatr. Dent..

[39] Arqub S.A., Al-Zubi K., Iverson M.G., Ioannidou E., Uribe F. (2022). The biological sex lens on early orthopaedic treatment duration and outcomes in Class III orthodontic patients: A systematic review. Eur. J. Orthod..

[40] Kakali L., Christopoulou I., Tsolakis I.A., Sabaziotis D., Alexiou A., Sanoudos M., Tsolakis A.I. (2021). Mid-term follow up effectiveness of facemask treatment in class III malocclusion: A systematic review. Int. Orthod..

